# VAR2CSA Serology to Detec*t Plasmodium falciparum* Transmission Patterns in Pregnancy 

**DOI:** 10.3201/eid2510.181177

**Published:** 2019-10

**Authors:** Ana Maria Fonseca, Raquel González, Azucena Bardají, Chenjerai Jairoce, Maria Rupérez, Alfons Jiménez, Llorenç Quintó, Pau Cisteró, Anifa Vala, Charfudin Sacoor, Himanshu Gupta, Jennifer Hegewisch-Taylor, Joe Brew, Nicaise Tuikue Ndam, Simon Kariuki, Marta López, Carlota Dobaño, Chetan E. Chitnis, Peter Ouma, Michael Ramharter, Salim Abdulla, John J. Aponte, Achille Massougbodji, Valerie Briand, Ghyslain Mombo-Ngoma, Meghna Desai, Michel Cot, Arsenio Nhacolo, Esperança Sevene, Eusebio Macete, Clara Menéndez, Alfredo Mayor

**Affiliations:** ISGlobal, Hospital Clinic–Universitat de Barcelona, Barcelona, Spain (A.M. Fonseca, R. González, A. Bardají, M. Rupérez, A. Jiménez, L. Quintó, P. Cisteró, H. Gupta, J. Hegewisch-Taylor, J. Brew, C. Dobaño, J.J. Aponte, C. Menéndez, A. Mayor);; ICBAS–Universidade do Porto, Porto, Portugal (A.M. Fonseca);; Centro de Investigação em Saúde da Manhiça, Maputo, Mozambique (R. González, A. Bardají, C. Jairoce, M. Rupérez, A. Vala, C. Sacoor, C. Dobaño, J.J. Aponte, A. Nhacolo, E. Sevene, E. Macete, C. Menéndez, A. Mayor);; Universidade Eduardo Mondlane, Maputo (E. Sevene); CIBER Epidemiología y Salud Pública, Madrid, Spain (R. González, A. Jiménez, A. Mayor, A. Bardají);; Université d’Aboméy Calavi, Cotonou, Benin (N.T. Ndam, A. Massougbodji);; Kenya Medical Research Institute, Kisumu, Kenya (S. Kariuki, P. Ouma);; BCNatal–Barcelona Center for Maternal-Fetal and Neonatal Medicine–Hospital Clínic and Hospital Sant Joan de Deu, Barcelona (M. López);; Vrije Universiteit Amsterdam, Amsterdam, the Netherlands (J. Brew);; MERIT, Institut de Recherche pour le Développement, Paris, France (N.T. Ndam, V. Briand, M. Cot);; Institut Pasteur, Paris (C.E. Chitnis);; Institute of Tropical Medicine, University of Tübingen, Tübingen, Germany (G. Mombo-Ngoma);; Bernhard Nocht Institute for Tropical Medicine, Hamburg, Germany (M. Ramharter, G. Mombo-Ngoma);; University Medical Center Hamburg-Eppendorf, Hamburg (M. Ramharter);; Ifakara Health Institute, Dar es Salaam, Tanzania (S. Abdulla);; Centre de Recherches Médicales de Lambaréné, Lambaréné, Gabon (G. Mombo-Ngoma);; Centers for Disease Control and Prevention, Atlanta, Georgia, USA (M. Desai)

**Keywords:** Malaria, pregnancy, serology, transmission, exposure, immunity, Plasmodium falciparum, parasites, VAR2CSA, Benin, Gabon, Mozambique, Kenya, Tanzania, Spain

## Abstract

Pregnant women constitute a promising sentinel group for continuous monitoring of malaria transmission. To identify antibody signatures of recent *Plasmodium falciparum* exposure during pregnancy, we dissected IgG responses against VAR2CSA, the parasite antigen that mediates placental sequestration. We used a multiplex peptide-based suspension array in 2,354 samples from pregnant women from Mozambique, Benin, Kenya, Gabon, Tanzania, and Spain. Two VAR2CSA peptides of limited polymorphism were immunogenic and targeted by IgG responses readily boosted during infection and with estimated half-lives of <2 years. Seroprevalence against these peptides reflected declines and rebounds of transmission in southern Mozambique during 2004–2012, reduced exposure associated with use of preventive measures during pregnancy, and local clusters of transmission that were missed by detection of *P. falciparum* infections. These data suggest that VAR2CSA serology can provide a useful adjunct for the fine-scale estimation of the malaria burden among pregnant women over time and space.

Agile malaria surveillance and response systems that can be sustained over time are needed for the optimal design of control programs (*1*,*2*). Rates of *Plasmodium falciparum* infection among pregnant women are sensitive to changes in transmission (*3*,*4*) and correlate well with infection in infants (*5*) and children (*6*,*7*). Thus, passive detection of malaria cases at maternal health care services constitutes a promising approach to providing contemporary data on the levels, and changes in levels, of malaria burden in the population for successful malaria control and elimination (*8*).

After exposure to *P. falciparum* parasites that sequester in the placenta (*9*), antibodies against VAR2CSA, a multidomain variant antigen of the *P. falciparum* erythrocyte membrane protein 1 family, develop in pregnant women (*10*). VAR2CSA is expressed on the surface of infected erythrocytes and mediates placental sequestration of parasites through binding to chondroitin sulfate A (*11*). Levels of antibodies against VAR2CSA are affected by variables that influence the risk for *P. falciparum* exposure (*12**–**14*) and mirror malaria trends during pregnancy (*3*). Moreover, levels of VAR2CSA IgG at delivery correlate with the risk for malaria in the offspring (*14*), suggesting the value of these antibodies for pinpointing areas of high malaria transmission (*15*). Because VAR2CSA antibodies persist after the infection is cleared (*16*), they can provide a sensitive adjunct for *P. falciparum* monitoring, especially in areas of low malaria endemicity, where the chances of detecting antibodies are higher than those of detecting the parasite (*17*).

The utility of serosurveillance depends mainly on specific properties of the antigen, including immunogenicity, polymorphism, cross-reactivity, and longevity of the antibodies. Because different VAR2CSA domains elicit IgG responses with varying magnitudes and dynamics (*16*,*18*,*19*), we hypothesized that short-lived antibodies against immunogenic nonpolymorphic VAR2CSA epitopes would enable a fine-scale estimation of recent *P. falciparum* transmission during pregnancy (*17*). We examined plasma from pregnant women living in areas in which *P. falciparum* transmission varied from high to low and absent (Benin, Gabon, Mozambique, Kenya, Tanzania, and Spain) against a quantitative suspension array containing VAR2CSA and general parasite antigens. We first selected IgG responses that were rapidly acquired after *P. falciparum* infection, did persist in circulation, and were sensitive to the level of parasite exposure in pregnant women from Mozambique and Spain. We then used the serologic assay to quantify the relationship of VAR2CSA antibody responses with *P. falciparum* infection as well as with temporal, spatial, and intervention-driven changes in malaria burden among pregnant women.

## Methods

### Study Sites, Population, and Procedures

We included in our study pregnant women who participated in 3 clinical trials of intermittent preventive treatment during pregnancy (IPTp) during 2003–2005 in Mozambique (NCT00209781) (*20*) and during 2010–2012 in Mozambique, Benin, Gabon, Kenya, and Tanzania (NCT00811421) (*21*,*22*). Participants were recruited at their first antenatal visit, and all received a long-lasting insecticide-treated bed net. During 2003–2005, all received 2 doses of sulfadoxine/pyrimethamine (*20*); during 2010–2012, they received 2 doses of mefloquine or sulfadoxine/pyrimethamine if they were not HIV infected (*21*) and 3 doses of mefloquine or placebo plus daily cotrimoxazol prophylaxis if they were HIV infected (*22*). At delivery, tissue samples from the maternal side of the placenta, as well as 50 μL peripheral and placental dried blood spots (DBS), were collected. Peripheral and placental blood from pregnant women in Mozambique and Benin were also collected into EDTA Vacutainer tubes (Becton Dickinson, https://www.bd.com) and centrifuged; plasma was stored at −20°C. From a subset of pregnant women in Mozambique who delivered during 2011–2012, peripheral blood samples were also collected at the first antenatal visit and before administration of the second IPTp dose. We geocoded the households of women in Mozambique by using a global information system. Clinical malaria episodes were treated according to national guidelines at the time of the study (*20**–**22*). DBS and plasma samples were also collected from 49 pregnant women never exposed to *P. falciparum* who delivered in 2010 at the Hospital Clinic of Barcelona (Barcelona, Spain).

The study was approved by the ethics committees from the Hospital Clínic of Barcelona, the Comité Consultatif de Déontologie et d’Éthique from the Institut de Recherche pour le Développement (Marseille, France), the Centers for Disease Control and Prevention (Atlanta, GA, USA), and national ethics review committees from each malaria-endemic country participating in the study. Written informed consent, which included permission to test for immune markers by using stored biological samples, was obtained from all participants.

### Laboratory Determinations

At recruitment, we assessed HIV serostatus by using rapid diagnostic tests according to national guidelines and hemoglobin level at delivery by using the following mobile devices on capillary blood samples: HemoCue (Danaher, http://www.hemocue.com), Hemocontrol (EKF Diagnostics, http://www.ekfdiagnostics.com), and KX analyzer (Sysmex, http://www.sysmex.com). Thick and thin blood films and placental biopsy samples were checked for *Plasmodium* spp. according to standard, quality-controlled procedures (*3*). We tested blood on filter paper for the presence of *P. falciparum* in duplicate by means of a real-time quantitative PCR (qPCR) targeting 18S ribosomal DNA (*3*).

### Antibody Measurements

We measured IgG in plasma (Benin and Mozambique) or on DBS (Gabon, Kenya, and Tanzania) in appropriate conditions for plasma elution (*19*) by using xMAP technology and the Luminex 100/200 System (https://www.luminexcorp.com) for 37% of pregnant women participating in the clinical trials with samples available. We constructed 2 multiplex suspension array panels (Appendix 1) (*19*), 1 including *P. falciparum* recombinant proteins (VAR2CSA Duffy binding-like recombinant domains DBL3X, DBL5Ɛ; and DBL6Ɛ, apical membrane antigen 1 [AMA1]; and 19-kDa fragment of the merozoite surface protein-1 [MSP1_19_], from 3D7 strain) and 1 consisting of synthetic peptides (25 VAR2CSA peptides covering conserved and semiconserved regions of VAR2CSA and a circumsporozoite peptide [pCSP]) (*19*). To assess unspecific IgG recognition, we used bovine serum albumin in both arrays (*19*). Procedures for reconstitution of DBS and quality control, bead-based immunoassay, data normalization, and definition of seropositivity cutoffs are described in Appendix 1.

### *var2csa* Sequencing and 3D Protein Modeling

We used DNA extracted from 50 DBS that were *P. falciparum* positive by qPCR for Sanger sequencing of *var2csa* PCR amplification products covering peptides of interest (Appendix 1). Sequence variability with respect to the peptide included in the array was assessed after amino acid alignment, and a 3D model of the DBL1X-ID1 region was developed by using Chimera version 1.5.3 (https://www.cgl.ucsf.edu); Appendix 1).

### Definitions and Statistical Analyses

We included in the analysis pregnant women for whom all information was available for IPTp, date of delivery, HIV status, age, parity, and antibody responses. We classified women as primigravid (first pregnancy) or multigravid (>1 previous pregnancy) and categorized age as <20, 20–24, or >25 years (*13*). Anemia was defined as hemoglobin level at delivery <11 mg/L. We compared proportions by using the Fisher exact test. We used univariate regression models to evaluate the association of log-transformed IgG levels (linear) and seropositivity (logistic) with study periods (2004–2005 and 2010–2012) and country, *P. falciparum* infection, parity, anemia, and IPTp intervention, taking into account potential confounding variables (HIV and age) in multivariate models. We assessed the modification of the associations by HIV infection or parity by including interaction terms into the regression models. To control the false discovery rate in the selection of antigens, we computed adjusted p values (q-values) by using the Simes procedure (*23*). We used multilevel mixed-effect linear regression analysis to estimate half-life and time to double (T_2×_) IgG levels in the longitudinal cohort of pregnant women from Mozambique (Appendix 1). We identified spatial clusters of *P. falciparum* infection and seropositivity as well as the most likely hotspots by using the Ward hierarchical cluster analysis and Kulldorff spatial scan method (Appendix 1). We performed statistical analyses by using Stata/SE software version 12.0 (StataCorp, https://www.stata.com), R statistics software version 3.2.1 (https://www.r-project.org), and Graphpad Prism version 6 (https://www.graphpad.com).

## Results

### Study Participants and *P. falciparum* Prevalence

Study participants consisted of 2,354 pregnant women (Table; Appendix 1 Figure 2) recruited during 2004–2005 (n = 146) and 2010–2012 (n = 2,208) in the context of IPTp clinical trials (*20**–**22*). Among them, 993 were from Mozambique, 854 from Benin, 131 from Gabon, 296 from Kenya, 31 from Tanzania, and 49 from Spain. The baseline characteristics of the women selected for this trial were similar to those of the 6,216 women participating in the randomized clinical trials (Appendix 2 Table 1).

**Table:** Participants in study of VAR2CSA serologic testing to detec*t Plasmodium falciparum* transmission patterns, by country and HIV status*

Variable	HIV-uninfected, no. (%)							HIV-infected, no. (%)			
	2004–2005		2010–2012					2004–2005		2010–2012	
	Mozambique, n = 65		Mozambique,† n = 485	Benin, n = 854	Gabon, n = 131	Tanzania, n = 31		Mozambique, n = 81		Mozambique,† n = 362	Kenya, n = 296
Parity											
Primigravid	17 (26)		181 (37)	188 (22)	38 (29)	16 (52)		28 (35)		46 (13)	22 (7)
Multigravid	48 (74)		304 (63)	666 (78)	93 (71)	15 (48)		53 (65)		316 (87)	274 (93)
Age, y											
<20	19 (29)		181 (37)	86 (10)	42 (32)	5 (16)		27 (33)		41 (11)	15 (5)
20–24	17 (26)		123 (25)	281 (33)	45 (34)	14 (45)		26 (32)		84 (23)	96 (32)
>25	29 (45)		181 (37)	487 (57)	44 (34)	12 (39)		28 (35)		237 (65)	185 (62)
IPTp											
Sulfadoxine/pyrimethamine	65 (100)		151 (31)	288 (34)	55 (42)	11 (35)		81 (100)		0	0
Mefloquine	0		334 (69)	566 (66)	76 (58)	20 (65)		0		178 (49)	139 (47)
Placebo‡	0		0	0	0	0		0		184 (51)	157 (53)
Microscopy§¶											
Positive	9 (14)		13 (3)	110 (15)	3 (2)	1 (3)		8 (10)		8 (2)	15 (5)
Negative	56 (86)		468 (97)	616 (85)	125 (98)	30 (97)		73 (90)		323 (98)	268 (95)
qPCR¶#											
Positive	16 (25)		28 (6)	332 (46)	9 (10)	0		21 (26)		13 (4)	22 (8)
Negative	49 (75)		424 (94)	393 (54)	80 (90)	31 (100)		60 (74)		314 (96)	251 (92)

*IPTp, intermittent preventive treatment during pregnancy; qPCR, quantitative PCR. †40% (196/485) of HIV-uninfected and 12% (43/362) of HIV-infected participants were pregnant women with samples collected also at recruitment and second IPTp administration. ‡All HIV-infected women who received placebo were also receiving cotrimoxazol prophylaxis. §Maternal microscopic infection defined by the presence of *P. falciparum* parasites in peripheral blood or in placenta on microscopic or histologic examination, respectively. ¶Not determined: 179 microscopy and 196 qPCR. #Maternal qPCR-positive infection was defined by a positive result on qPCR testing in peripheral or placental blood.

The study areas represented 5 sites in sub-Saharan Africa with different intensities of malaria transmission. Prevalence of *P. falciparum* infection detected by qPCR at delivery, in either peripheral or placental blood (averaged for 2010–2012), among HIV-uninfected women was 46% (332/725) in Benin, 10% (9/89) in Gabon, and 6% (28/452) in Mozambique and among HIV-infected women was 8% (22/273) in Kenya and 4% (13/327) in Mozambique (Table). The prevalence of *P. falciparum* infection among pregnant women in Mozambique decreased from 25% (37/146) in 2004–2005 to 2% (3/176) in 2010 and increased to 6% (4/72) in 2012. A subset of 239 pregnant women from Mozambique recruited during 2011–2012 was followed during pregnancy; prevalence of *P. falciparum* infection detected by qPCR was 16% (38/239) at first antenatal visit (mean gestational age ± SD, 20.7 ± 5.45 weeks), 3% (8/239) at the second IPTp administration (25.9 ± 4.98 weeks), and 5% (13/239) at delivery (38.4 ± 2.26 weeks). *P. falciparum* infection was detected at unscheduled visits for 2% (5/239) of the women. Overall, *P. falciparum* infection was detected at any of these time points for 21% (49/239) of the women.

### *P. falciparum*–Specific Antibody Profiles and Parasite Exposure during Pregnancy

**Figure 1:**
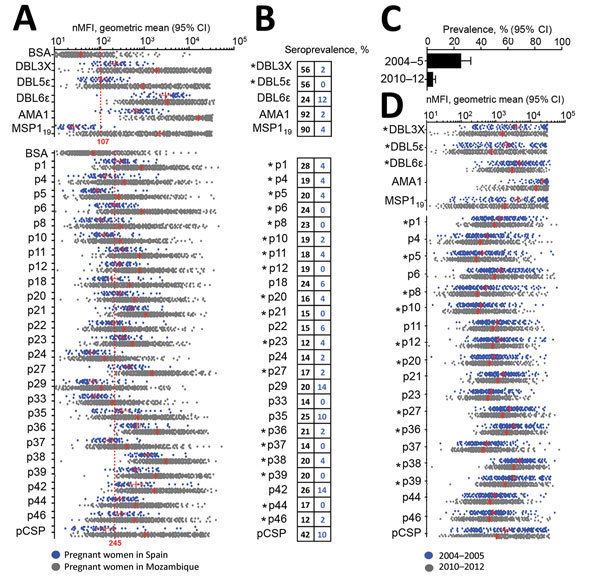
*Plasmodium falciparum* VAR2CSA IgG in malaria-exposed and -nonexposed pregnant women. A) nMFI measured in pregnant women from Mozambique and Spain. Red dashed line represents the mean nMFI from bovine serum albumin + 3 SDs. B) Seroprevalence among pregnant women from Spain (blue) and Mozambique (black). Asterisks indicate antigens recognized by pregnant women from Mozambique at levels above IgG against bovine serum albumin + 3 SDs and above levels in pregnant women from Spain (q-value <0.05 by Simes procedure) and those antigens poorly recognized by pregnant women from Spain (seroprevalence <5%). C, D) Prevalence of *P. falciparum* infection in peripheral and placental blood by quantitative PCR (C) and nMFIs (D) among pregnant women from Mozambique delivering in 2004–2005 and 2010–2012. Red lines represent the geometric mean and T-bars the 95% CI. Asterisks indicate antigens recognized by IgG whose levels dropped between 2004 and 2012, as assessed by linear regression adjusted by intermittent preventive treatment during pregnancy, parity, age, and HIV status (q-value <0.05 by Simes procedure). nMFI, normalized median fluorescent intensity.

Mean antiparasite IgG levels in pregnant women from Mozambique delivering from 2010 through 2012 were above levels against bovine serum albumin plus 3 SD and higher than IgG levels in pregnant women from Spain except for DBL6Ɛ and 3 of 25 VAR2CSA peptides (Figure 1, panel A; Appendix 2 Table 3). Five VAR2CSA peptides, DBL6Ɛ, and pCSP were recognized by IgG from >5% of the pregnant women from Spain who had never been exposed to *P. falciparum* (Figure 1, panel B), suggesting unspecific recognition; thus, these peptides were excluded from subsequent analysis. To further narrow down the VAR2CSA peptide candidates, we compared IgG levels in pregnant women from Mozambique delivering in 2004–2005 and 2010–2012, a period when *P. falciparum* prevalence assessed by qPCR at delivery in peripheral or placental blood dropped from 25% to 5% (Figure 1, panel C) (*3*). This decline in infection rates was mirrored by drops of IgG levels against 10 of the 18 previously selected VAR2CSA peptides (p1, p5, p8, p10, p12, p20, p27, p36, p38, p39) (Figure 1, panel D; Appendix 2 Table 4).

### Acquisition and Decay of IgG Responses against VAR2CSA

**Figure 2:**
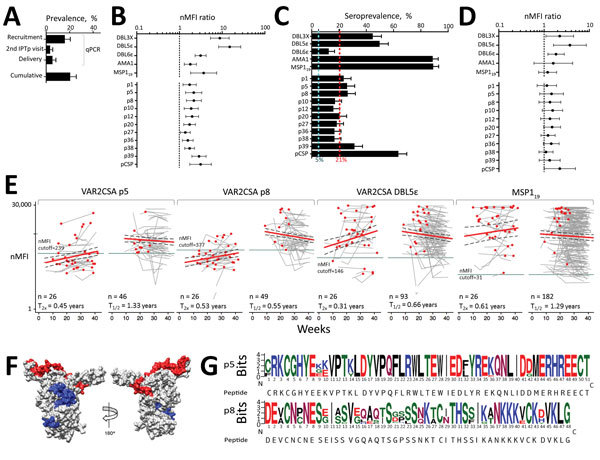
IgG responses during pregnancy against selected VAR2CSA antigens and polymorphism in target sequences in serologic study of *Plasmodium falciparum *in pregnant women. A) *P. falciparum* prevalence by quantitative PCR (qPCR) in 239 pregnant women from Mozambique at recruitment, second administration of IPTp, and delivery. Cumulative prevalence at delivery refers to peripheral or placental infection detected by microscopy, qPCR, or histology at any time point. B) Ratio of nMFIs at delivery in women from Mozambique infected during pregnancy compared with uninfected women. Error bars indicate 95% CIs. C) Seroprevalence at delivery, showing the cumulative prevalence of infection during pregnancy (red dashed line) and the prevalence at delivery by qPCR (light blue line). D) Ratio of nMFIs at delivery in multigravid compared with primigravid women, adjusted by IPTp, parity, age, and HIV status. Error bars indicate 95% CIs. E) IgG dynamics during pregnancy with estimates of time to double (T_2x_) and half-life (T_1/2_) obtained from linear mixed-effect regression model. Red points represent *P. falciparum* infection, dark gray lines the seropositivity cutoff, red lines the fitted-estimation, and dashed lines the 95% CI. F) Space-feeling representation of DBL1X-ID1 showing p5 (blue) and p8 (red). G) Logo representation of p5 and p8 sequences obtained from 50 *P. falciparum* isolates (20 from Mozambique, 10 from Benin, 10 from Gabon, and 10 from Kenya). IPTp, intermittent preventive treatment during pregnancy; nMFI, normalized median fluorescent intensity.

We assessed the dynamics of IgG responses in a longitudinal cohort of 239 pregnant women from Mozambique (Figure 2, panel A). At delivery, compared with uninfected women, the 49 (21%) women infected with *P. falciparum* during pregnancy had higher IgG levels against the 10 down-selected peptides (Figure 2, panel B; Appendix 2 Table 5). At delivery, seroprevalence rates for p1 (23%), p5 (26%), p8 (26%), and p39 (31%) antibodies were above the cumulative prevalence of *P. falciparum* infection during pregnancy (Figure 2, panel C; Appendix 2 Table 5). No difference in IgG levels was observed between primigravid and multigravid women (Figure 2, panel D; Appendix 2 Table 5). T_2×_ after *P. falciparum* infection ranged from 0.45 years (95% CI 0.31–0.80 years) for p5 to 1.07 years (95% CI 0.60–5.23 years) for p27 (Figure 2, panel E; Appendix 2 Table 6). IgG half-life among seropositive women at recruitment without evidence of *P. falciparum* infection during follow-up ranged from 0.55 (95% CI 0.38–1.02) years for p8 to 3.66 (95% CI 0.98–∞) years for p1 (Figure 2, panel E; Appendix 2 Table 6). Among recombinant antigens, IgG DBL5Ɛ showed the lowest T_2×_ (0.31 [95% CI 0.21–0.61] years) and half-life (0.66 [95% CI 0.42–1.65] years), whereas AMA1 IgG showed the highest T_2×_ (1.76 [95% CI 0.76–∞] years) and half-life (4.18 [95% CI 1.86–∞] years).

**Figure 3:**
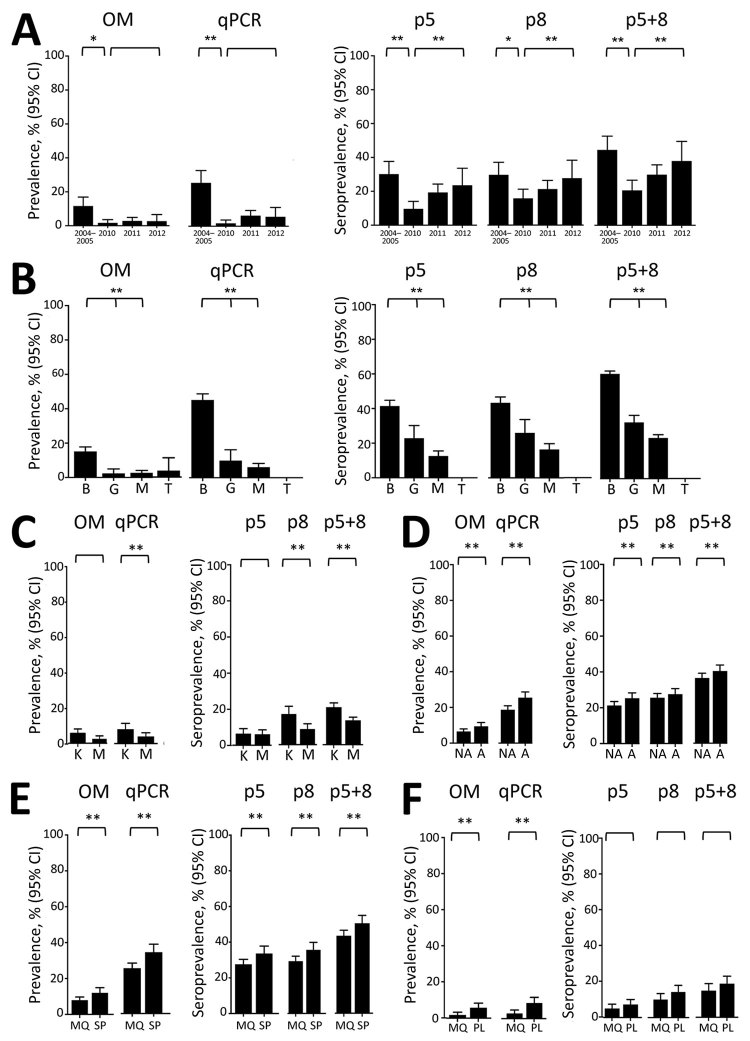
IgG seroprevalence against VAR2CSA selected antigens according to study period, country, anemia status and intermittent preventive treatment group in the serological study of *Plasmodium falciparum* in pregnant women. A) Pregnant women from Mozambique delivering during different periods. B) HIV-uninfected pregnant women from Benin, Gabon, Mozambique, and Tanzania. C) HIV-infected pregnant women from Kenya and Mozambique. D) Nonanemic (NA) and anemic (A) pregnant women. E) HIV-uninfected pregnant women receiving mefloquine (MQ) or sulfadoxine/pyrimethamine (SP) as intermittent preventive treatment during pregnancy (IPTp). F) HIV-infected pregnant women receiving MQ or placebo (PL) as IPTp. Maternal microscopic infection was defined by the presence of *P. falciparum* parasites in peripheral blood or in placenta on microscopic or histologic examination, respectively. Maternal quantitative PCR (qPCR)–positive infection was defined by a positive result on qPCR of peripheral or placental blood. P-values were obtained from multivariate regression models adjusted for HIV, parity, age, IPTp, and country when applicable. T-bars represent 95% CIs. *Crude p<0.05; **adjusted p<0.05. B, Benin; G, Gabon; K, Kenya; M, Mozambique; OM, optical microscopy; T, Tanzania.

**Figure 4:**
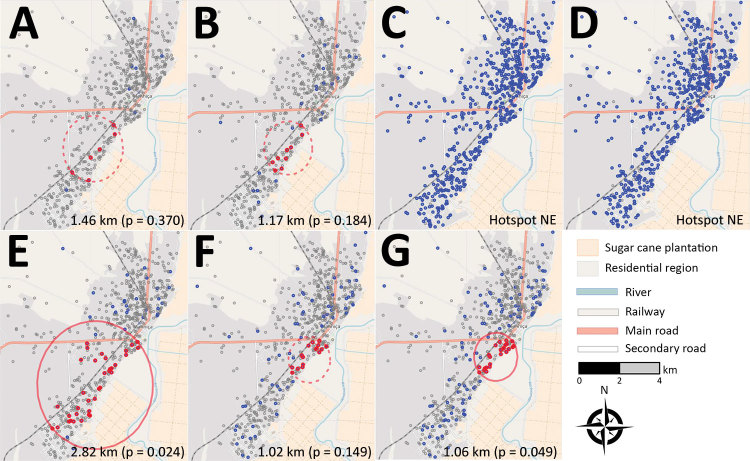
Geographic patterns of *Plasmodium falciparum* infection and IgG seropositivity in pregnant women living in southern Mozambique. Geographic distribution of seropositive pregnant women (HIV-uninfected and HIV-infected) living in Manhiça District, Mozambique, who delivered during 2010–2012 and for whom microscopy, quantitative PCR (qPCR), and spatial geocoordinates were available. Distribution shows pregnant women with and without *P. falciparum* infection at delivery, either in peripheral or in placental blood, detected by microscopy or histology (A) or qPCR (B), as well as AMA1 (C), MSP1_19_ (D), p5 (E), p8 (F), and p5+8 (G) seropositive and seronegative pregnant women at delivery. Grey dots indicate seronegative women, blue dots indicate seropositive women, red dots indicate seropositive women selected by the hotspot cluster algorithm; red circles indicate the most likely hotspot (continuous line p<0.05, dashed line p>0.05). Maps were generated by using OpenStreetMap (https://www.openstreetmap.org). Hotspot NE, not estimated because of low/high prevalence of seroresponders.

Among the down-selected VAR2CSA peptides (p1, p5, p8, and p39), IgG against p5 (51 amino acids) and p8 (48 amino acids) showed the lowest half-lives (0.55 [95% CI 0.38–1.02] years for p8; 1.33 [95% CI 0.65–∞] years for p5) and the largest increase in women exposed to *P. falciparum* during pregnancy compared with uninfected women (adjusted ratio [AR]_p5_ 2.15 [95% CI 1.39–3.31] and AR_p8_ 2.17 [95% CI 1.46–3.23]; Figure 2, panel B; Appendix 1 Figure 5; Appendix 2 Table 5). IgG levels and seroprevalence rates at delivery for p5 and p8 were higher among pregnant women with active or past malaria infection than among women with no parasite or pigment in the placenta, as assessed by histologic examination (Appendix 2 Table 7). 3D modeling mapped both sequences on the exposed surface of DBL1X-ID1 region of VAR2CSA (Figure 2, panel F). Amino acid variability obtained from 50 *P. falciparum* isolates collected at study sites was 5% ± 2 SD for p5 sequences and 16% ± 5 SD for p8 sequences, compared with the consensus peptide sequence included in the array (Figure 2, panel G; Appendix 1 Figures 3, 4).

### Performance of Selected VAR2CSA Peptides for Assessing Spatial and Temporal Differences in *P. falciparum* Exposure

In pregnant women from Mozambique at delivery, p5 and p8 seroprevalence rates, as well as the composite of both (p5+8), decreased from 2004–2005 to 2010 (adjusted odds ratio [AOR]_p5+8_ 0.27 [95% CI 0.11–0.68]), followed by an increase from 2010 to 2012 (AOR_p5+8_ 2.49 [95% CI 1.34–4.61]; Figure 3, panel A; Appendix 2 Table 8). This decrease and subsequent increase mirrored *P. falciparum* prevalence by qPCR. HIV infection and parity did not modify the associations observed (p value for interaction >0.05 for all cases; Appendix 2 Table 8). Similar to *P. falciparum* prevalence determined by qPCR, seroprevalence rates were the highest in HIV-uninfected women from Benin, followed by those from Gabon (AOR_p5+8_ 0.31 [95% CI 0.21–0.47]) and Mozambique (AOR_p5+8_ 0.21 [95% CI 0.16–0.28]; Figure 3, panel B; Appendix 2 Table 9). At delivery, pregnant women living in an area from Tanzania where no *P. falciparum* infection was detected by qPCR were seronegative for p5, p8, and p5+8 antibodies; 42% were seropositive against AMA1 and 48% were seropositive against MSP1_19_ antibodies (Figure 3, panel B). Among HIV-infected women, seroprevalence rates for p8 and p5+8 were lower in Mozambique than in Kenya (AOR_p5+8_ 0.58 [95% CI 0.38–0.88]; Figure 3, panel C; Appendix 2 Table 9). p5 and p5+8 seroprevalence rates were higher among anemic than among nonanemic women (AOR_p5+8_ 1.26 [95% CI 1.03–1.55]; Figure 3, panel D; Appendix 2 Table 10). Seroprevalence rates were lower among HIV-uninfected women who received IPTp with mefloquine than among those who received sulfadoxine/pyrimethamine (AOR_p5+8_ 0.74 [95% CI 0.59–0.94]; Figure 3, panel E; Appendix 2 Table 11). Seroprevalence rates among HIV-infected women were lower among those who received mefloquine than among those who received placebo, although differences were not significant (AOR_p5+8_ 0.76 [95% CI 0.50–1.15]; Figure 3, panel F; Appendix 2 Table 11).

### Geographic Patterns of *P. falciparum *Transmission through VAR2CSA Serologic Testing

Spatial geocoordinates were available for 698 pregnant women from Mozambique residing in Manhiça District (southern Mozambique). Geographic areas experiencing significantly higher seroprevalence rates than would be expected by chance were observed for p5 (radius 2.82 km; p = 0.024) and p5+8 (radius 1.06 km; p = 0.049) but not for MSP1_19_ and AMA1 (Figure 4; Appendix 2 Table 12). The distribution of HIV infection, parity, age, and IPTp was similar among women inside and outside the serologic hotspot (p>0.05; Appendix 2 Table 12).

## Discussion

Routine *P. falciparum* testing of easily accessible pregnant women at maternal healthcare services has the potential to offer a rapid, consistent, and cost-effective method for evaluating the malaria burden in different communities and tracking progress of interventions. IgGs against 2 VAR2CSA peptides, selected according to their ability to maximize the information about recent *P. falciparum* exposure during pregnancy, reflected differences in malaria burden over time and space in multiple settings in Africa and changes in parasite rates associated with the use of different preventive regimens. Overall, our results indicate that in areas with well-attended maternal healthcare services, this pregnancy-specific serologic test may serve as a useful sentinel surveillance tool for flagging changes in malaria burden and progress in the path toward elimination.

p5 (51 amino acids) is localized in the DBL1X domain and p8 (48 amino acids) in the ID1 region of VAR2CSA. Limited diversity (5%) of p5 sequence was observed in *P. falciparum* isolates from a variety of regions of Africa, in accordance with estimates from previous studies for the DBL1X domain (*24*). p8 corresponds to a more diverse (16%) variant of the ID1 region in VAR2CSA (*25*). Both peptides are exposed on the DBL1X-ID1 N terminal region of VAR2CSA (*18*,*26*) and recognized by IgG from malaria-exposed pregnant women at levels higher than those of pregnant women from Spain and men from Mozambique (*19*). IgG responses against both VAR2CSA peptides increased with *P. falciparum* infection during pregnancy. Moreover, higher risk for anemia among p5 and p5+8 seroresponders support these antibodies as markers of recent infection, which adversely affects the women’s health (*3*). In contrast to the slow decay of IgG responses against AMA1, the half-life of IgG against p5 and p8 was <2 years, the average time reported in Mozambique for a second pregnancy to occur (*27*). The short half-life of p5 and p8 IgG, together with the similar IgG levels in multigravid and primigravid women, suggests that antibodies acquired during one pregnancy are not maintained over multiple pregnancies; thus, antibodies can be used as a reliable indicator of recent exposure for pregnant women, regardless of parity.

Seroprevalence rates for p5, p8, and the composite of both peptides (p5+8) mirrored trends in *P. falciparum* prevalence among pregnant women from Mozambique delivering during 2004–2012 (*3*), a temporal pattern that was also observed for PfPR_2–10_ (*28*). Trends were similar among HIV-uninfected and infected women, suggesting that impairment of *P. falciparum*–specific antibody responses driven by viral infection (*29*) may not affect short-lived IgG responses against p5 and p8. Seroprevalence also reflected the burden of malaria among pregnant women residing in a variety of settings in Africa, as well as reductions in infection rates resulting from the use of mefloquine as IPTp among HIV-uninfected women (*21*). Similar trends, although not statistically significant, were observed among HIV-infected women receiving cotrimoxazol prophylaxis alone or in combination with mefloquine (*22*), possibly because of the longer duration of protection provided by 3 IPTp doses in HIV-infected women compared with the 2 doses in HIV-uninfected women. We also found that pregnant women living in an area from Tanzania where no *P. falciparum* infection was detected by qPCR as well as pregnant women from Spain never exposed to malaria were seronegative against p5 and p8, suggesting that pregnancy-specific serology might be used to confirm the eventual interruption of transmission.

Geographic distribution of pregnant women from Mozambique who were seropositive against p5 and p5+8 revealed a serologic hotspot in an area close to the river and sugar cane plantations, where the density of anopheline mosquitoes can be expected to be higher. In contrast, antibodies against MSP1_19_ and AMA1 were not able to identify these malaria transmission patterns because of saturation of antibody responses after lifelong exposure to *P. falciparum*. These results support the value of using VAR2CSA serologic testing to amplify signals of recent exposure and suggest its potential to trigger targeted interventions to persons living in close proximity to passively detected seropositive pregnant women.

Our study has several limitations. First, the peptide array we used may have missed some conformational nonlinear epitopes. Second, different transmission dynamics and host genetic factors may affect the acquisition and decay of antibodies (*16*). Third, steeper decay of antibodies may be observed out of pregnancy when infecting parasites express non-VAR2CSA variants. Fourth, the reduction of data from median fluorescence intensity to seroprevalence to simplify the serologic information of the assay may reduce the depth of serologic information. Developing alternative mathematical models that use antibody levels (*30*) may increase the sensitivity to detect temporal and spatial changes in malaria transmission. Fifth, small numbers of pregnant women from malaria-free areas in Tanzania and Spain limit the generalizability of our data to support pregnancy-specific serologic testing as a tool to confirm interruption of transmission. Last, antibody assessments in this study were conducted mainly at delivery; further studies should assess the performance of this testing at antenatal visits or soon after delivery (i.e., during infant immunization). Future research is needed to describe the relationship between pregnancy-specific serologic testing and malaria transmission in the general population and its value for confirming interruption of malaria transmission and providing early signals of *P. falciparum* resurgence after local elimination.

In summary, this study shows that IgG against 2 VAR2CSA peptides from the DBL1X-ID1 domain reveal temporal and spatial differences in malaria burden among pregnant women and reductions in exposure associated with the use of preventive measures during pregnancy. These antibodies enable the identification of local clusters of transmission that are missed by detection of *P. falciparum* infections. Our results suggest that inferring recent exposure through VAR2CSA serologic testing would amplify signals of ongoing malaria transmission and increase the power to detect changes, either natural or driven by deliberate efforts, as well as malaria hotspots, among pregnant women (*2*). Moreover, peptides such as p1 targeted by long-lasting IgG responses may be useful for capturing past changes in transmission by sampling women of child-bearing age and relating seroprevalence with the number and timing of previous pregnancies. Operationally suitable serologic tests (*31*) capable of detecting antibodies against VAR2CSA synthetic peptides may be used in programmatic environments to stratify areas based on malaria burden, measure the effects of interventions, and document year-to-year changes in transmission. 

Appendix 1 Supplemental methods and results from study of VAR2CSA serologic testing to detect *Plasmodium falciparum* transmission patterns.

Appendix 2 Supplemental results from study of VAR2CSA serologic testing to detect *Plasmodium falciparum* transmission patterns.
