# Supplementary material for: VAR2CSA Serology to Detect Plasmodium falciparum Transmission Patterns in Pregnancy
Source: Emerg Infect Dis. 2019 Oct;25(10):1851–60. doi: 10.3201/eid2510.181177 (PMC6759269; doi:10.3201/eid2510.181177)
Supplement: Appendix 1 — Supplemental methods and results from study of VAR2CSA serologic testing to detect Plasmodium falciparum transmission patterns. [file 18-1177-Techapp-s1.pdf]

Article DOI: https://doi.org/10.3201/eid2510.181177

VAR2CSA Serology to Detect *Plasmodium falciparum* Transmission Patterns

Appendix 1

P. falciparum antigens

A total of 46 peptides (length between 35 and 65 aminoacids) from conserved and semi-conserved regions of the VAR2CSA protein were designed after alignment by Clustal W of 18 VAR2CSA full-length sequences from *P. falciparum* isolates of different geographic origins (Asia, Africa, Central and South America) (*1*). The 25 peptides analyzed (3 peptides from DBL1X, 3 from DBL2X, 5 from DBL3X, 2 from DBL4Ɛ, 5 from DBL5Ɛ, 2 from DBL6Ɛ and 5 from NTS and ID regions) were previously selected based on highly recognized by IgGs from plasma of malaria exposed pregnant women compared with Spanish individuals and malaria exposed Mozambican men (*1*) (Appendix Table 2). A circumsporozoite peptide (pCSP) of 64 aminoacids (NVDP[NANP]$_{15}$) was also analyzed. Peptides were synthetized by Gl Biochem (Xangai, China) and median purity was estimated as 79% (range: 71%–91%) by high performance liquid chromatography (HPLC) and mass spectrometry. The Duffy binding-like recombinant domains (DBL3X, DBL5Ɛ and DBL6Ɛ) and the *P. falciparum* general antigens (AMA1 and MSP1$_{19}$) were all produced at ICGEB, New Delhi, India. *Clostridium tetani,* tetanus toxin was purchased from Santa Cruz Biotechnology (Dallas, Texas) to control for the amount of IgGs eluted from dried blood spots (DBS).

Bead-based immunoassay

Two multiplex suspension array panels were constructed to quantify IgG responses against *P. falciparum* recombinant proteins and synthetic peptides, using the xMAP technology and the Luminex® 100/200 System (Luminex® Corp., Austin, Texas). MagPlex® microspheres (magnetic carboxylated polystyrene microparticles, 5.6 µm) with different spectral signatures were selected for each protein (DBL3X, DBL5Ɛ, DBL6Ɛ, AMA1 and MSP1$_{19}$), peptide (25

VAR2CSA peptides and pCSP), tetanus toxin and bovine serum albumin. Antigens were covalently coupled to beads following a modification of the Luminex® Corporation protocol (*1*). Briefly, 200 µl of beads (2,5x10^6) were transferred into a 1.5 mL eppendorf tube and resuspended by sonication and vortexing. The supernatant was removed after precipitation of the beads by magnetic separation during 60 seconds. Beads were washed twice with 250 µl of distilled water and pellets were resuspended in 80 µl of activation buffer (0.1 M NaH2PO4, pH 6.2). Sulfo-NHS (N-hydroxysulfosuccinimide) and EDC (1-Ethyl- 3-[3-dimethylaminopropyl] carbodiimide hydrochloride; Pierce, Thermo Fisher Scientific Inc., Rockford, IL) were simultaneously added to reaction tubes at 5 mg/mL each in activation buffer, and reaction tubes were incubated at room temperature with gentle agitation, protected from light for 20 minutes. Activated beads were washed twice with 250 µl of coupling buffer (MES 50 mM, 2-[N-morpholino] ethanesulfonic acid monohydrate pH 5, Sigma-Aldrich). Antigen amounts in the coupling reaction for one million beads were determined after a titration experiment, and were as follows: 2 µg of tetanus toxin, 4 µg of DBL3X, DBL5Ɛ, DBL6Ɛ, AMA1, 8 µg of $MSP1_{19}$, 170 µg of each peptide and for bovine serum albumin a 1% solution in PBS (Phosphate-buffered saline) and volume was adjusted with coupling buffer until a maximum of 400 µl. Beads and antigens were vortexed, sonicated and then incubated over night at 4°C in the dark, with shaking. Coupled beads were blocked with 500 µl of 1% BSA in PBS for 30 minutes on a shaker at room temperature, avoiding light and then washed twice with 500 µl of assay buffer (1% BSA, 0.05% sodium azide in PBS filtrated) and resuspended in 400 µl of the same buffer. Beads were quantified on a Guava PCA desktop cytometer (Guava, Hayward, CA), and stored at 4°C in the dark. Protein and peptide multiplex arrays were prepared by pooling together equal volumes of coated beads.

Immediately before use, stock suspensions of antigen-coated microspheres were thoroughly resuspended by vortexing and sonication. Frozen plasma or the product of DBS elution were thawed at room temperature, mixed by vortexing, and spun at 16000 g for 5 minutes to remove particles. 50 µl of diluted microspheres (1000 microspheres/analyte/well) were added to a 96-well Mylar flat-bottom plate following the addition of 50 µl of diluted sample in duplicates to a final concentration of 1:400 for protein array and 1:100 for peptide array and incubated for 1 hour in agitation, protected from light at room temperature. After incubation, the plates were washed three times with 200 µl of washing buffer (0.05% Tween 20 in PBS) by

pelleting in a magnetic 96 well separator. 100 µl of biotinylated anti-human IgG (Sigma, Tres Cantos, Spain) diluted 1:1000 in assay buffer was added to each well, and plates were incubated for 45 minutes in agitation, protected from light at room temperature. After the incubation period, the plates were washed and 100 µl of streptavidin-conjugated R-phycoeryhtrin (Invitrogen, Carlsbad, CA) at a 1:1000 dilution in assay buffer was added and incubated for 25 minutes. Finally the plates were washed as before and the beads were resuspended in 100 µl of assay buffer and analyzed using the Luminex® 100/200 System.

A hyperimmune plasma pool composed by 23 plasmas from *P. falciparum* infected Mozambican pregnant women was tested to determine if the coupling was effective and was included in each assay plate as positive control, in addition to blanks (wells without sample) to assess background levels. A minimum of 50 microspheres were read per spectral signature and results were exported as crude median fluorescent intensity (MFI). Duplicates were averaged and background MFIs were subtracted. A total of 224 plates were analyzed and the intra-assay variation (mean CV of replicates from 20 plasma samples per plate) ranged from 1.4% to 7.3% for the protein array and from 2.5% to 12.4% for the peptide array. The inter-assay variation (variability of positive pool between 224 plates) was 5% for the protein array and 26% for the peptide array (*1*). To assure the validity of the luminex plates, a quality control was performed on the MFI values of the positive control pool. Results were plotted in Levey Jenning Charts (Appendix Figure 1) and five plates from the peptide array fell out of –2 standard deviation (SD) and +2SD and were re-analyzed (*2*). Results were normalized (nMFI) to account for plate-to-plate variation by dividing the background subtracted MFI of each sample by the value of the positive pool in the same plate and multiplying by the median of positive pools in all plates.

Definition of IgG seropositivity

Seropositivity cutoffs were obtained using finite mixture models (FMM) for pregnancy-specific malaria antigens (VAR2CSA peptides and recombinant domains). FMM can be applied in scenarios of heterogeneous IgG distributions where a subgroup of the study population is seronegative and another seropositive avoiding additional sampling of malaria never exposed pregnant women. However, seropositivity cutoffs to general malaria antigens (AMA1, $MSP1_{19}$ and pCSP) were obtained from means plus three SD of IgG levels from never exposed pregnant

women as a consequence of absence of heterogeneous IgG distribution against *P. falciparum* general antigens in malaria exposed adult pregnant women (*1*).

Reconstitution of dried blood spots

Antibodies were eluted from a total of 880 DBS from Gabon (n = 310), Kenya (n = 408) and Tanzania (n = 162) as previously described (*1,3*). Briefly, four spots of ≈3 mm in diameter were cut from the filter papers using a punch (McGill® round punch, 3 mm) and transferred to individual wells of a 96-well polystyrene U-bottom plate. Antibodies were eluted with 200 µl Luminex® assay buffer (1% BSA, 0.05% sodium azide in filtrated PBS) at room temperature overnight with gentle mixing which, assuming a hematocrit of 50%, gives a concentration of eluted blood proteins equivalent to a 1:50 plasma dilution (*3,4*).

To assess the quality of the elution, hemoglobin levels in the eluted DBS were measured by spectrophotometry (wavelengths 415, 380 and 450) and calculated using the Harboe method with the Allen correction (Hb [mg/l] = 167.2 x A415 – 83.6 x A380 – 83.6 x A450) x dilution factor). Three criteria to discard DBS improperly eluted were followed, as previously described (*1*). First, 259 reddish-brown spots against a pale background were discarded after visual examination of reconstituted spots.(*3*) Second, 10 DBS were also discarded because hemoglobin levels measured in the elutions were below the upper quartile of hemoglobin value among samples considered with inappropriate visual aspect (hemoglobin upper quartile = 7.4m/l). Finally, 153 samples were also discarded because anti-tetanus antibodies measured in the elutions were below the lowest quartile obtained from anti-tetanus IgG among samples with appropriate visual aspect and hemoglobin levels (anti-tetanus lowest quartile = [11563,5 nMFI]). Finally, 458/880 samples (131 from Gabon, 296 from Kenya and 31 from Tanzania) were considered as correctly eluted.

Sequencing of *var2csa* p5 and p8 in *P. falciparum* isolates

A total of 50 *P. falciparum* isolates collected on filter paper (Whatman 903) from infected individuals in Mozambique (n = 20), Benin (n = 10), Gabon (n = 10) and Kenya (n = 10) were selected for DNA sequencing. A half of the filter paper containing a 25 µL of blood drop was used for DNA extraction using a QIAamp DNA Mini kit (Qiagen), as per the manufacturer's

instructions. Finally, DNA was eluted in 100 µL of AE buffer given in the kit. The presence of *P. falciparum* infection was detected using a previously described method (*5,6*).

We designed a single polymerase chain reaction (PCR) based assay to amplify purified DNA templates using 2720 Thermal Cycler (Applied Biosystems) followed by Sanger sequencing for *var2csa* gene. In brief, a 25 µl reaction was set up, containing 0.5 µM of each forward (p5_F- 5′aaggtgtgggaagttattac-3′) and reverse (p8_R- 5′attagttaaagatgcaagtact-3′) primers, 1x HOT FirePol Master Mix (Solis BioDyne; Cat. No. 04–27–00125) and 5 µl of template DNA. The reaction volume was raised by PCR-grade water. The template DNA was denatured at 95°C for 15 minutes in a thermocycler, followed by 35 cycles of amplification (95°C for 1 minute, 54°C for 1 minute, and 72°C for 1 minute) and a final extension at 72°C for 10 minutes. A reaction using 5 µl of PCR-grade water instead of template DNA was included as a negative control. PCR products were run on 1.5% agarose (Invitrogen) gels in 1× TBE buffer (Tris/Borate/EDTA; Thermo Fisher Scientific) to determine the presence and size of the amplified DNA. PCR products were visualized using a ultraviolet trans-illuminator. The PCR primer set was also tested with human gDNA to check their specificity. The expected size of the PCR was 960 bp covering amino acid positions 220 to 539 of *var2csa* gene. PCR products were quantified using EPOCH Biotech system. Approximately 1200 ng of PCR products were sent to Genewiz, following safety instructions for the accurate shipment of PCR amplicons. To sequence the p5 and p8 fragments of *var2csa* gene, three new sequencing primers were used (Seq_p5_R - 5′ccatttcttcacacattcac-3′; Seq_p8_F - 5′gggtgatccttatttcgcagaa-3′; Seq_p8_R- 5′cgcaagaaatcttggcaacaaca-3′) along with PCR p5_F primer. This allowed us to sequence 960 bp *var2csa* amplicons in both directions. The bi-directional sequencing with PCR p5_F and Seq_p5_R, and Seq_p8_F and Seq_p8_R primer sets covered 220–335 and 389–499 aa respectively.

The variations in the test sequences were identified by sequence alignment (Blastn, NCBI:https://blast.ncbi.nlm.nih.gov) against reference sequence of 3D7 (PF3D7_1200600) retrieved from PlasmoDB. The nucleotide sequences obtained from field isolates were translated using ExPASy online tool (http://web.expasy.org/translate/) and represented as a logo figure using the Weblogo Version 2.8.2 online tool (http://weblogo.berkeley.edu).

VAR2CSA DBL1X-ID1 3D model

The 3D-structure of DBL1X-ID1 was obtained by submitting the 3D7 sequence, with domain limits defined by Bockhorst and colleagues (2007) (*7*) to the HHPred server (http://toolkit.tuebingen.mpg.de/hhpred). The structure with highest HHPred score, corresponding to the DBL1α domain of the VarO strain (Protein Data Bank [PDB] 2yk0 (*8*)), was selected for homology modeling in MODELER based on the default alignment. Molecular graphics were generated in UCSF Chimera version 1.5.3 (*9*).

IgG dynamic analysis through mixed-effects regression models

Summary statistics according to the longitudinal design were calculated by trimester. Time-at-risk was estimated using gestational age at recruitment as the time when subjects first came under observation, and gestational age at delivery as the latest time under which the subjects were both under observation and at risk. Antibody levels were analyzed assuming a lognormal distribution, and therefore they were described by the geometric means and the overall, between and within-subjects standard deviations.

The crude and adjusted effect of *P. falciparum* infection on antibody levels was analyzed using log-linear mixed-effects regression models incorporating Gaussian random intercepts. This resulted in an estimate of the rates of antibody dynamics (boosting or decay), assuming a single exponential model. Time to 2-fold increase were calculated from the estimated rates and the boundaries at 95% confidence interval (CI) obtained from mixed-effects models for women with *P. falciparum* infection at follow-up (*10,11*). Similarly, half-lives were calculated from models including women who were seropositive at recruitment with no *P. falciparum* infection at follow-up (*10,11*). Where the boosting rate is a negative value (rate below 1) or the decay rate is a positive value (rate above 1), the calculated time to 2-fold increase or half-life was reported as infinity. Statistical comparisons were performed at two-sided significance level of 0.05 and 95% CI were calculated for all estimations.

Consider our longitudinal dataset consisting of antibody measurements of 239 pregnant women on three successive gestational ages. Because we were not really interested in these particular 239 women per se, we treated them as a random sample from a larger population and

modeled the between-woman variability as a random effect, as a random-intercept term at the woman level. We thus fitted the model:

$$\ln(C_{ij}) = \beta_0 + \beta_1 T_{ij} + \upsilon_j + \varepsilon_{ij}$$

where C_{ij} are the concentrations for i = 1 .. Three measurement of gestational age (T) and j = 1 .... 239 women. The fixed portion of the model, $\beta_0 + \beta_1 T_{ij}$, simply states that we wanted one overall regression line representing the population average. The random effect υ_j serves to shift this regression line up or down according to each woman. Back-transforming the measurements to the original scale we obtain the following overall regression line:

$$C_{ij} = e^{\beta_0 + \beta_1 T_{ij}} = e^{\beta_0} e^{\beta_1 T_{ij}} = C_0 e^{\beta_1 T_{ij}}$$

where C_0 is the baseline concentration. We can estimate the time required to obtain a value Δ times C_{ij}

$$\Delta C_{ij} = C_0 e^{\beta_1 (T_{ij} + t_\Delta)} = C_0 e^{\beta_1 T_{ij}} e^{\beta_1 t_\Delta} = C_{ij} e^{\beta_1 t_\Delta}$$

and therefore

$$\Delta = e^{\beta_1 t_\Delta}$$

$$\ln(\Delta) = \beta_1 t_\Delta$$

$$t_\Delta = \ln(\Delta) / \beta_1$$

In particular, for half-life Δ = 1/2 and then $t_{1/2} = \ln(1/2) / \beta_1 = -\ln(2) / \beta_1$. Similarly, time to 2-fold increase can be calculated ad $t_{2x} = \ln(2) / \beta_1$.

Geospatial analysis

Spatial hotspots of *P. falciparum* infection and seropositivity among pregnant women from Mozambique living in Manhiça district were designated using hierarchical cluster analysis with Ward's minimum variance method (*12*). Kulldorff spatial scan method (*13–15*) was used to identify the most likely hotspot for *P. falciparum* infection and seropositivity, setting our significance threshold (α) as 0.05. Hotspots of greater size than one cluster were identified by consecutively aggregating nearest-neighboring areas until a proportion of the total study population was included. To ascertain statistical significance, we employed Monte Carlo sampling, using Poisson likelihoods, and following the original Kulldorff method for

identification of clusters (*16*). Analysis were performed using the R statistical software (version 3.2.1) (*17*) and maps were generated using OpenStreetMap (*18*). The key R packages used were SpatialEpi (*19*), deldir (*20*), geosphere (*21*), rgeos (*22*) raster (*23*) and leaflet (*24*).

References

1. Fonseca AM, Quinto L, Jiménez A, González R, Bardají A, Maculuve S, et al. Multiplexing detection of IgG against *Plasmodium falciparum* pregnancy-specific antigens. PLoS One. 2017;12:e0181150. PubMed https://doi.org/10.1371/journal.pone.0181150

2. Kerkhof K, Canier L, Kim S, Heng S, Sochantha T, Sovannaroth S, et al. Implementation and application of a multiplex assay to detect malaria-specific antibodies: a promising tool for assessing malaria transmission in Southeast Asian pre-elimination areas. Malar J. 2015;14:338. PubMed https://doi.org/10.1186/s12936-015-0868-z

3. Corran PH, Cook J, Lynch C, Leendertse H, Manjurano A, Griffin J, et al. Dried blood spots as a source of anti-malarial antibodies for epidemiological studies. Malar J. 2008;7:195. PubMed https://doi.org/10.1186/1475-2875-7-195

4. Stewart L, Gosling R, Griffin J, Gesase S, Campo J, Hashim R, et al. Rapid assessment of malaria transmission using age-specific sero-conversion rates. PLoS One. 2009;4:e6083. PubMed https://doi.org/10.1371/journal.pone.0006083

5. Mayor A, Serra-Casas E, Bardají A, Sanz S, Puyol L, Cisteró P, et al. Sub-microscopic infections and long-term recrudescence of Plasmodium falciparum in Mozambican pregnant women. Malar J. 2009;8:9. 10.1186/1475-2875-8-9 PubMed https://doi.org/10.1186/1475-2875-8-9

6. Taylor SM, Mayor A, Mombo-Ngoma G, Kenguele HM, Ouédraogo S, Ndam NT, et al. A quality control program within a clinical trial Consortium for PCR protocols to detect *Plasmodium* species. J Clin Microbiol. 2014;52:2144–9. PubMed http://dx.doi.org/10.1128/JCM.00565-14

7. Bockhorst J, Lu F, Janes JH, Keebler J, Gamain B, Awadalla P, et al. Structural polymorphism and diversifying selection on the pregnancy malaria vaccine candidate VAR2CSA. Mol Biochem Parasitol. 2007;155:103–12. PubMed http://dx.doi.org/10.1016/j.molbiopara.2007.06.007

8. Vigan-Womas I, Guillotte M, Juillerat A, Hessel A, Raynal B, England P, et al. Structural basis for the ABO blood-group dependence of *Plasmodium falciparum* rosetting. PLoS Pathog. 2012;8:e1002781. PubMed https://doi.org/10.1371/journal.ppat.1002781

9. Pettersen EF, Goddard TD, Huang CC, Couch GS, Greenblatt DM, Meng EC, et al. UCSF Chimera—a visualization system for exploratory research and analysis. J Comput Chem. 2004;25:1605–12. PubMed http://dx.doi.org/10.1002/jcc.20084

10. Wipasa J, Suphavilai C, Okell LC, Cook J, Corran PH, Thaikla K, et al. Long-lived antibody and B Cell memory responses to the human malaria parasites, *Plasmodium falciparum* and *Plasmodium vivax*. PLoS Pathog. 2010;6:e1000770. PubMed http://dx.doi.org/10.1371/journal.ppat.1000770

11. Kinyanjui SM, Conway DJ, Lanar DE, Marsh K. IgG antibody responses to *Plasmodium falciparum* merozoite antigens in Kenyan children have a short half-life. Malar J. 2007;6:82. PubMed https://doi.org/10.1186/1475-2875-6-82

12. Murtagh F, Legendre P. Ward's hierarchical clustering method: clustering criterion and agglomerative algorithm. J Classif. 2011;31:274–95. 10.1007/s00357-014-9161-z https://doi.org/10.1007/s00357-014-9161-z

13. Kulldorff M. Spatial scans statistics: models, calculations, and applications. Scan Statistics and Applications. 1999;303–22.

14. Lawson AB, Kleinman KP. Spatial and syndromic surveillance for public health. West Sussex, England (UK); John Wiley & Sons; 2005.

15. Lin P-SK, Kung YH, Clayton M. Spatial scan statistics for detection of multiple clusters with arbitrary shapes. Biometrics. 2016;72:1226–34. PubMed https://doi.org/10.1111/biom.12509

16. Kulldorff M, Nagarwalla N. Spatial disease clusters: detection and inference. Stat Med. 1995;14:799–810. PubMed https://doi.org/10.1002/sim.4780140809

17. Kusi KA, Bosomprah S, Dodoo D, Kyei-Baafour E, Dickson EK, Mensah D, et al. Anti-sporozoite antibodies as alternative markers for malaria transmission intensity estimation. Malar J. 2014;13:103. PubMed https://doi.org/10.1186/1475-2875-13-103

18. Ramm JF, Topf J, Chilton S. *OpenStreetMap: Using and Enhancing the Free Map of the World.* Cambridge, UK: UIT Cambridge Limited; 2010).

19. Kim AY, Wakefield J. SpatialEpi: methods and data for spatial epidemiology. R package version 1.2.2 [cited 2017 Nov 5]. https://CRAN.R-project.org/package=SpatialEpi

20. Turner R. Deldir: Delaunay triangulation and Dirichlet (Voronoi) tessellation. R package version 0.1–14 [cited 2017 Nov 5]. https://CRAN.R-project.org/package=deldir, (2017).

21. Hijmans RJ. Geosphere: spherical trigonometry. R package version 1.5–5 [cited 2017 Nov 5]. https://CRAN.R-project.org/package=geosphere, (2016).

22. Bivand R, Rundel C. rgeos: interface to geometry engine - open source (GEOS). R package version 0.3–19 [cited 2017 Nov 5]. https://CRAN.R-project.org/package=rgeos, (2016).

23. Hijmans RJ. raster: Geographic Data Analysis and Modeling. R package version 2.5–8 [cited 2017 Nov 5]. https://CRAN.R-project.org/package=raster, (2016).

24. Cheng J, Karambelkar B, Xie Y. Leaflet: create interactive Web maps with the JavaScript Leaflet Library. R package version 1.1.0 [cited 2017 Nov 5]. https://CRAN.R-project.org/package=leaflet, (2017).

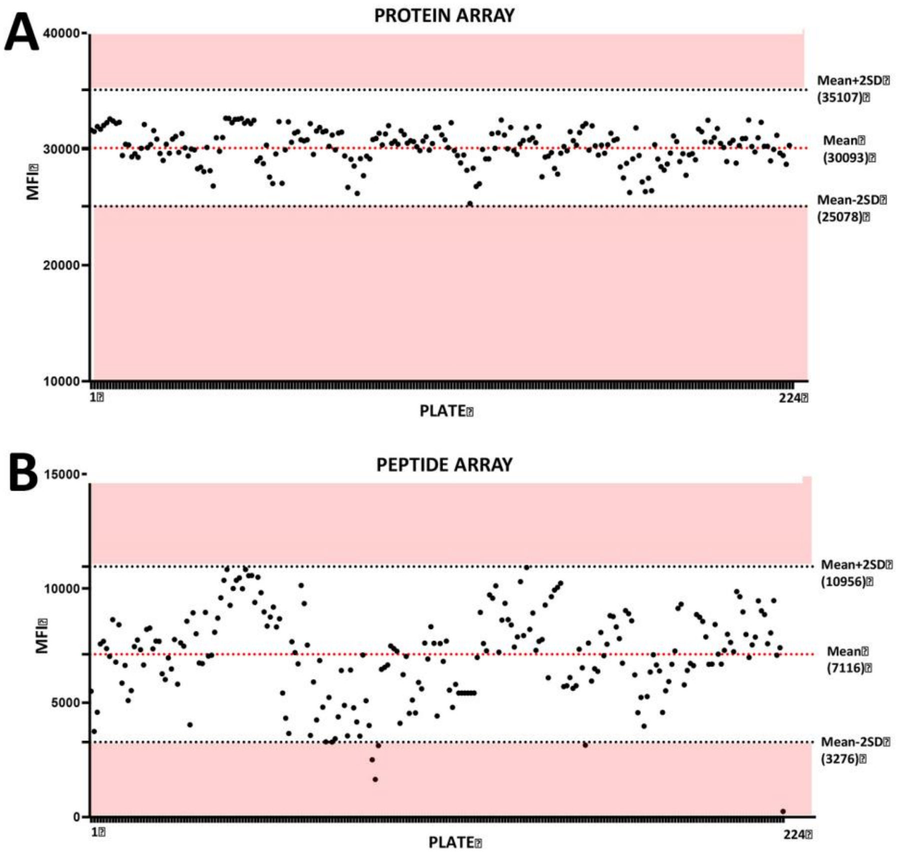

Appendix Figure 1. Levey Jenning Charts plotted for the quality control of the Luminex assay. Data analysis for a quality control of the MFI values obtained from the hyperimmune plasma pool (positive pool) for the A) protein array and B) peptide array. The dots represent each positive pool per plate. If these dots fell out of the mean of positive pool +/– two standard deviation (SD) (red area), these plates were rejected and re-analyzed.

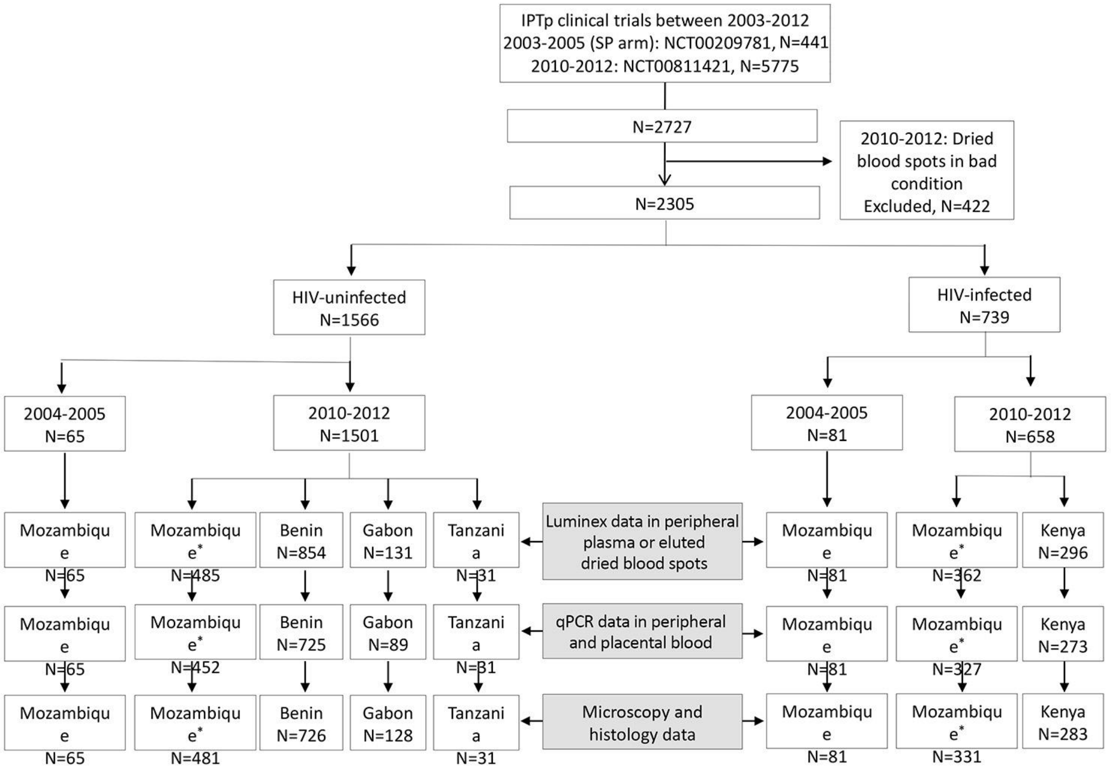

Appendix Figure 2. Study profile. *40% (196/485) of HIV-uninfected and 12% (43/362) of HIV-infected women participated in a longitudinal study in which samples were collected at recruitment, second IPTp administration and delivery (data available for qPCR, microscopy and histology).

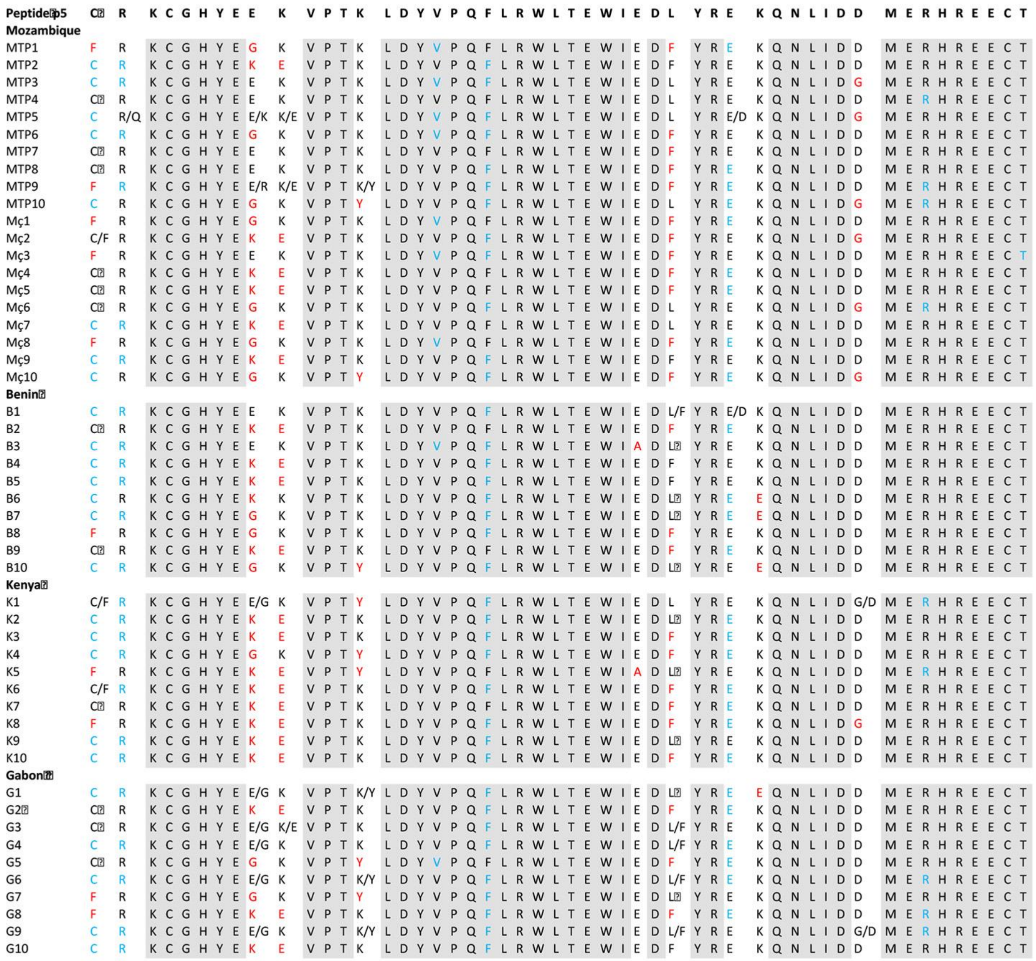

Appendix Figure 3. VAR2CSA peptide p5 sequences from *P. falciparum* isolates from Mozambique, Benin, Kenya and Gabon. Grey fill means conserved regions, red indicate aminoacid change, blue indicate nucleotide change but same aminoacid and positions with two aminoacids correspond to mixed infections. Samples Mç1–10, B1–10, K1–10 and G1 were collected from pregnant women in Mozambique (Mç), Benin (B), Kenya (K) and Gabon (G) who were participating in the clinical trial (2010–2012). Samples MTP1–10 we obtained from Northern Mozambique (Montepuez).

Sample	Sequence
**Peptide p8**	**DEVCNCNESEISSVGQAQTSGPSSNKTCITHSSIKANKKKVCKDVKLG**
**Mozambique**	
MTP1	DEVCNCNESEISSVEQAQTSRPSSNKTCITHSSIKANKKKVCKDVKLG
MTP2	DEVCNPNESEISSVGQAQTSGSLSQKACNTHSSIKANKKKVCKDVKLG
MTP3	DEVCNPNESGIASVEHAQTSGSSSNKTCITHSSIKTNKKKVCKHVKLG
MTP4	DEVCNPNESGIASVEHAQTSGSSSNKTCITHSSIKTNKKKVCKHVKLG
MTP5	DEACNCNESGISSVEQAQTSDPSSNKTCNTHSSIKANKKKECKDVKLG
MTP6	DEVCNCNESEISSVEQEQISGPSSNKTCNTHSSIKANKKKVCKHVKLG
MTP7	DEVCNCNESGIASVGQASISDRSSQKACITHSFIGANKKKVCKDVKLG
MTP8	DEACNCNESEISSVGQASISDSSSQKACNTHSFIKTNKKKECKDVKLG
MTP9	DEACNPNESEISSVGQASISDSSSQKACNTHSFIKTNKKKECKDVKLG
MTP10	DEACNPNESGIASVEHAQTSGSSSNKTCITHSSIKTNKKKVCKHVKLG
Mç1	DEVCNPNESGISSVGQASTSCPSSNKTCITHSSIKANKKKVCKDVKLG
Mç2	DEVCNPNESEISSVGQAQTSDPSSNKTCNTHSSIKANKKKVCKHVKLG
Mç3	DEVCNPNESEISSVEQAQTSDPSSNKTCNTHSSIKANKKKVCKHVKLG
Mç4	DEACNCNESEIASVGQEQISDSSSNKTCNTHSSIKTNKKKVCKHVKLG
Mç5	DEVCNCNESEIASVGQASISDPSSNKTCNTHSSIKANKKKVCKDVKLG
Mç6	DEACNPNESGIASVEHAQTSGSSSNKTCITHSFIKTNKKKVCKDVKLG
Mç7	DEVCNCNESGIACVELAPISDSSSQKACITHSSIKANKKKVCKDVKLG
Mç8	DEVCNPNESGISSVGQAQTSCPSSNKTCITHSSIKANKKKVCKDVKLG
Mç9	DEVCNPNESEISSVGQAQTSGSLSQKACNTHSSIKANKKKVCKDVKLG
Mç10	DEACNPNESEISSVEQASISGSSSNKTCNTHSSIKTNKKKECKDVKLG
**Benin**	
B1	DEACNCNESEIASVGQEQISGPSSNKTCNTHSFIGANKKKVCKYVKLG
B2	DEVCNCNESGISSVGQAQTSGSSSNKTCITHSSIKTNKKKECKDVKLG
B3	DEVCNPNESEISSVEHAQTSVLLSQKAYITHSSIKANKKKVCKYVKLG
B4	DEVCNPNESEISSVGQAQTSGSLSQKACNTHSSIKANKKKVCKDVKLG
B5	DEVCNPNESEISSVGQAQTSGSLSQKACNTHSSIKANKKKVCKDVKLG
B6	DEVCNPNESEIACVELAQTSDSSSQKACITHSSIKANKKKVCKDVKLG
B7	DEVCNCNESGIASVGQAQTSDALSQKACITHSFIGANKKKECKDVKLG
B8	DEACNCNESGISSVELEQTSCPSSNKTCITHSSIKTNKKKVCKHVKLG
B9	DEVCNCNESGISSVGQAQTSDSSSNKTCITHSSIKTNKKKECKDVKLG
B10	DEVCNPNESEIASVGLAQTSVLLSQKAYITHSSIKANKKKVCKYVKLG
**Kenya**	
K1	DEVCNCNESKIACVELAPISDPSSNKTCITHSSIKANKKKVCKHVKLG
K2	DEVCNCNESGISSVELAQTSCPSSNKTCITHSSIKTNKKKVCKDVKLG
K3	DEVCNCNESGISSVELAQTSCPSSNKTCITHSSIKTNKKKVCKDVKLG
K4	DEACNPNESEIASVEQASISGSSSNKTCNTHSFIKANKKKVCKDVKLG
K5	DEACNCNESEIASVEQAQTSGPSSNKTCITHSSIKTNKKKVCKHVKLG
K6	DEVCNPNESGIASVELAQTSDPSSNKTCITHSSIKANKKKVCKHVKLG
K7	DEACNCNESKIASVGQEQISGSSSNKTCNTHSSIKTNKKKECKYVKLG
K8	DEVCNPNESGIASVEQAQTSDPSSNKTCTTHSSIKANKKKVCKHVKLG
K9	DEACNPNESGIACVGQAQTSGPSSNKTCITHSFIGANKKKVCKDVKLG
K10	DEVCNCNESGISSVELAQTSCPSSNKTCITHSSIKTNKKKVCKDVKLG
**Gabon**	
G1	DEACNPNESGIACVGQAQTSGSSSNKTCITHSSIKANKKKECKHVKLG
G2	DEVCNCNESEIASVEQAPISDRSSQKACITHSSIKTNKKKVCKHVKLG
G3	DEVCNPNESGIASVGQESISDRSSQKACITHSFIGANKKKVCKDVKLG
G4	DEACNCNESEIASVGQEQISGPSSNKTCNTHSFIGANKKKVCKYVKLG
G5	DEACNPNESGISSVEQEQISGPSSNKTCITHSFIKANKKKVCKDVKLG
G6	DEACNPNESEIASVEQASISGSSSNKTCNTHSFIKANKKKVCKDVKLG
G7	DEVCNPNESGISSVELAQISDRLSQKACNTHSSIKANKKIVCKHVKLG
G8	DEACNCNESEISSVGQAQISGSSSNKTCNTHSSIKTNKKKECKDVKLG
G9	DEACNPNESEIASVEQAPISGSSSNKTCNTHSFIKANKKKVCKDVKLG
G10	DEVCNPNESEISSVGQAQTSGSLSQKACNTHSSIKANKKKVCKDVKLG

Appendix Figure 4. VAR2CSA peptide p8 sequences from *P. falciparum* isolates from Mozambique, Benin, Kenya and Gabon. Grey fill means conserved regions, red indicate aminoacid change and blue indicate nucleotide change but same aminoacid. Samples Mç1–10, B1–10, K1–10 and G1 were collected from pregnant women in Mozambique (Mç), Benin (B), Kenya (K) and Gabon (G) who were participating in the clinical trial (2010–2012). Samples MTP1–10 we obtained from Northern Mozambique (Montepuez).

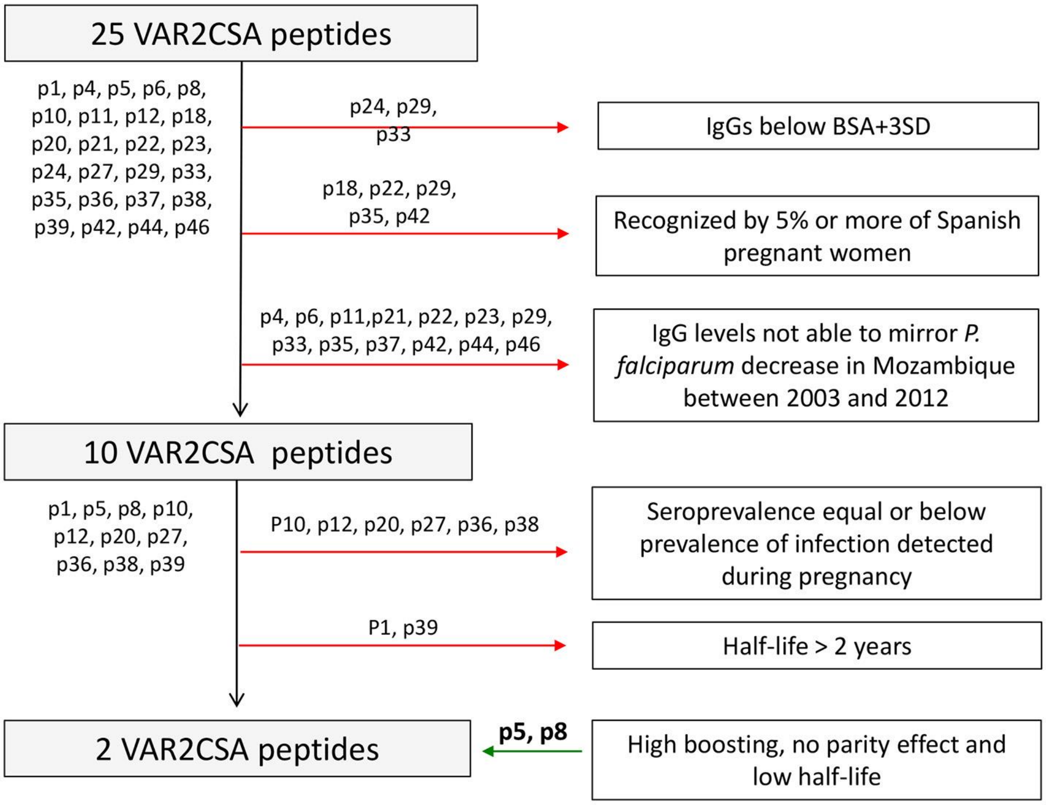

Appendix Figure 5. Diagram of peptide selection.
